# Epithelioid angiomyolipoma of the liver: CT and MRI features

**DOI:** 10.1007/s00261-012-9911-5

**Published:** 2012-05-20

**Authors:** Jian-song Ji, Chen-ying Lu, Zu-fei Wang, Min Xu, Jing-jing Song

**Affiliations:** 1 Department of Radiology, Lishui Central Hospital, Southeast University, 289 Kuocang Road, Lishui, 323000 Zhejiang China; 2 Department of Radiology, Lishui Central Hospital, Wenzhou Medical College, 289 Kuocang Road, Lishui, 323000 Zhejiang China

**Keywords:** Epithelioid angiomyolipoma, Liver, Computed tomography (CT), Magnetic resonance imaging (MRI)

## Abstract

The purpose of our study was to retrospectively evaluate the computed tomography (CT)/magnetic resonance imaging features of epithelioid angiomyolipoma of the liver (Epi-HAML), with pathology as a reference. We reviewed the CT/MRI findings of six lesions of Epi-HAML and found absence of adipose tissue in the lesions. In addition, recognizing the imaging features of no capsule, and hypervasularity with central punctiform or filiform vessels as a characteristic enhancement may distinguish Epi-HAML from other hepatic tumors.

Angiomyolipoma (AML) of the liver is an uncommon benign tumor, with abundant fatty component that can be easily diagnosed by CT or MRI. However, epithelioid AML of the liver (Epi-HAML) is a rare tumor of unpredictable behavior, that contains no or only a minimal amount of adipose tissue and is difficult to distinguish from other hypervascular tumors [1–3]. Rare reports of Epi-HAML diagnosed by CT and MRI are in the literature. In this study, we analyzed six lesions of Epi-HAML by CT and MRI, and described the imaging features of the tumor in order to achieve a better understanding of this disease.

## Subjects and methods

All the six patients who had Epi-HAML were women, aged 32–64 years with a median of 47 years. Epi-HAML lesions had been examinated by CT/MRI because of abdominal pain (*n* = 1), suspected gallbladder stone (*n* = 1) or on routine health examination (*n* = 4). Of the six patients, none had a history of cirrhosis or viral hepatitis and none had tuberous sclerosis. All patients were negative for HbsAg and a-fetoprotein (AFP) levels were within normal ranges.

Spiral CT scans were performed in four patients with Siemens Sensation 16-detect CT scanner. MRI was performed in two patients with Siemens Sensation 1.5-T magnetic resonance scanner. Contrast agents (80–100 mL) or Gd-DTPA (gadolinium diethylene triamine penta-acetic acid) of 15 mL were injected intravenously as a bolus, a triphasic contrast-enhanced dynamic exploration during the arterial, portal venous, and delayed phases was performed.

All patients received liver biopsy or partial hepatic resection. CT images of the four patients and MRI images of the two patients were available for retrospective analysis and were compared with pathological findings by three experienced radiologists.

## Results

Helical CT and MR imaging demonstrated an obvious tumor in all patients. The location, size, fat-containing, capsule, non-enhanced images as well as the enhanced images are listed in Table 1. Four lesions were located in the left lobe of the liver, one lesion was located in the right lobe and one in the caudate lobe. The lesions ranged from 5.0 to 9.5 cm in diameter. All masses were well-defined and no fatty attenuation or tense was found on CT or MRI. Late enhancement of a discrete capsule was observed in one mass.

**Table 1:** The location, size, fat-containing, capsule, unenhanced images and enhanced images of six cases of epithelioid hepatic AML (Epi-HAML)

Case no.	Sex	Age	Location	Size (cm)	Fat	Capsule	Vessels in lesion	Unenhanced CT/MR	Enhanced CT/MR arterial portal delayed		
1	F	64	S1	6.0	No	No	Yes	Hypo	Hyper	Hypo	Hypo
2	F	43	S2, S3	7.0	No	No	Yes	Hypo	Hyper	Iso	Iso
3	F	56	S4	5.0	No	No	No	Iso	Hyper	Hyper	Hyper
4	F	40	S2, S3	9.5	No	No	Yes	Hypo	Hyper	Hypo	Hypo
5	F	46	S7, S8	5.2	No	No	Yes	T1 hypo, T2 hyper	Hyper	Hypo	Hypo
6	F	32	S2, S3	7.0	No	Yes	Yes	T1 hypo, T2 hyper	Hyper	Hypo	Hypo

Cases 1, 2, 3, and 4 underwent CT examination, whereas cases 5 and 6 underwent MRI examination

*S* segment, *Hyper* hyperattenuation/hyperintense, *Hypo* hypoattenuation/hypointense, *Iso* isoattenuation, *T1 hypo* hypointense on T1WI, *T2 hyper* hyperintense on T2WI

**Fig. 1:**
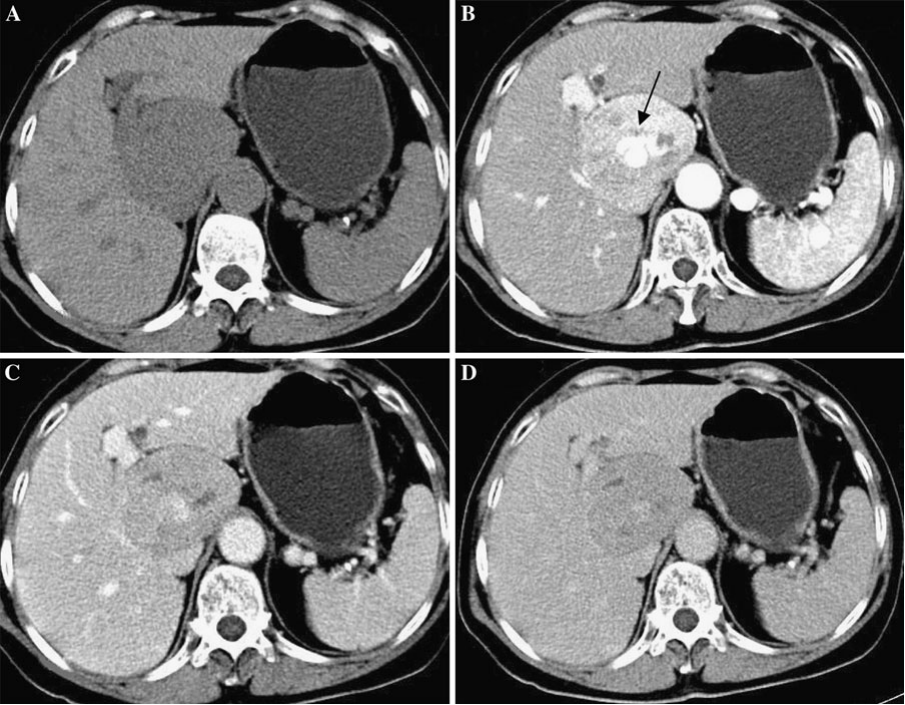
64-Year-old woman with epithelioid AML in caudate lobe of liver (patient 1). **A** Non-enhanced CT scan shows hypoattenuating lesion in segment. **B** Contrast-enhanced CT scan shows inhomogeneous enhanced lesion with thickly distorted vessels (*arrow*) in the arterial phase. **C**, **D** The lesion is hypoattenuating in the portal venous/delayed phase.

**Fig. 2:**
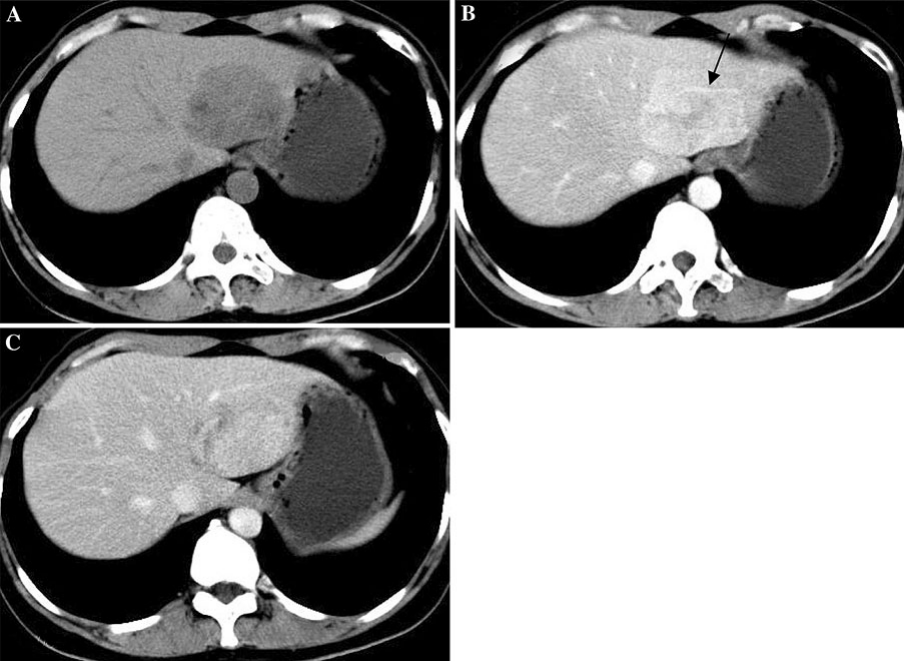
43-Year-old woman with epithelioid AML in left lobe of liver (patient 2). **A** Non-enhanced CT scan shows hypoattenuating lesion in segment 2/3. **B** Contrast-enhanced CT scan shows obviously enhanced lesion in the arterial phase; filiform vessels (*arrow*) within the lesion is show in the arterial phase image. **C** The lesion is isoattenuating in the portal venous phase.

**Fig. 3:**
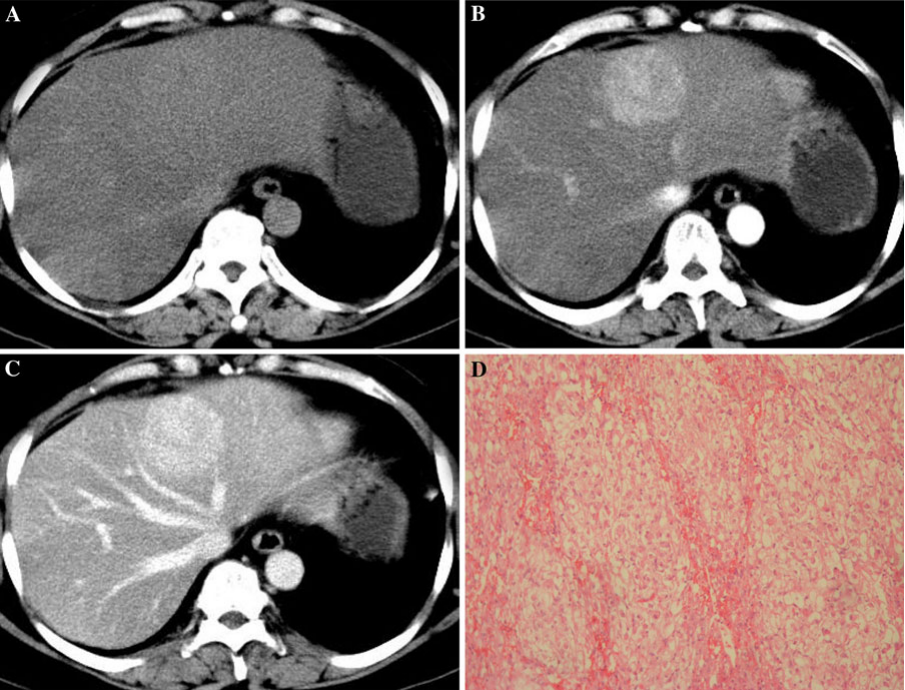
56-Year-old woman with epithelioid AML in left lobe of liver (patient 3). **A** Non-enhanced CT scan shows isoattenuating lesion in segment 4 with fatty liver disease. **B** Contrast-enhanced CT scan shows obviously enhanced lesion in the arterial phase. **C** The lesion shows prolonged hyperattenuating in the portal venous phase. **D** Microscopic examination shows that the tumor is almost exclusively composed of epithelioid cells.

With regard to the four patients who underwent CT, Epi-HAML was predominantly hypoattenuating to liver on unenhanced images in three patients (Figs. 1A, 2A), and isoattenuating in one patient with fatty liver disease (Fig. 3A). At hepatic arterial phase CT, the masses were heterogeneously hyperattenuating in all patients. Central punctiform or filiform vessels could be seen in three lesions(*arrows*) (Figs. 1B, 2B). Thick and curved vessels were present in two lesions. At portal venous and delayed phase CT, two lesions with abundant central vessels were hypoattenuating (Fig. 1C, D). One lesion with small vessels was isoattenuating (Fig. 2C), whereas another lesion with fatty liver disease without vessels was hyperattenuating (Fig. 3C).

**Fig. 4:**
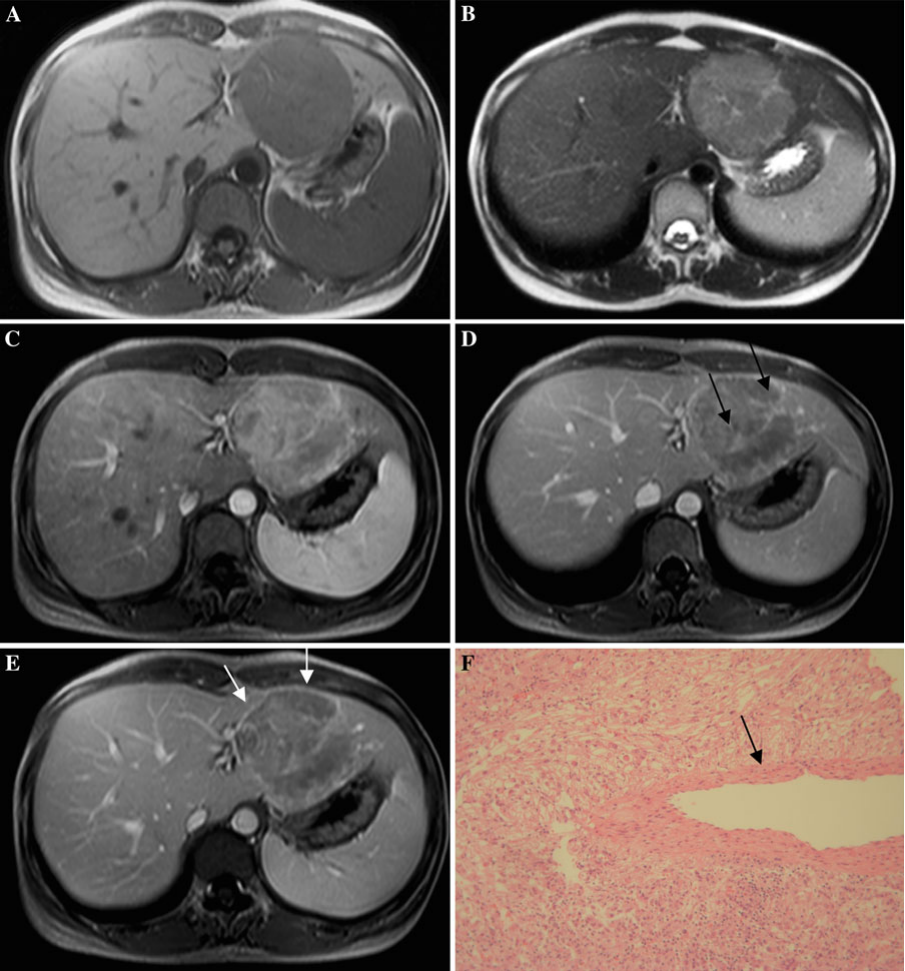
32-Year-old woman with epithelioid AML in left lobe of liver (patient 6). **A**, **B**: MRI shows a well-defined tumor, hypointense on T1-weighted and hyperintense on T2-weighted images in segment 2/3. **C**–**E**: Contrast-enhanced MRI shows inhomogeneous enhanced lesion with punctiform or filiform vessels (*arrows*) and discrete capsule enhancement (*white arrows*). **F** Microscopically, the tumor consists of epithelioid cells with no fat component. There is a thick-walled blood vessel in the center (*arrow*).

With regard to the two patients who underwent MRI, the lesions were hypointense on T1WI and hyperintense on T2WI (Fig. 4A, B). On hepatic arterial phase T1-weighted MR images, tumors were heterogeneously hyperintense to liver in both patients (Fig. 4 **C**). On portal venous and delayed phase T1-weighted MR images, the two tumors were hypointense to liver (Fig. 4D, E). Central punctiform and filiform vessels could be seen in both lesions (*arrows*) and discrete capsule enhancement was evident in one lesion (white arrows) (Fig. 4B–E).

Histologically, the tumor was almost exclusively composed of epithelioid cells, with little fat and thick-walled blood vessels (Figs. 3D, 4F). All the tumor cells demonstrated positive immunostaining for HMB-45.

## Discussion

AML is an uncommon tumor that is composed of blood vessels and mature fat cells as well as smooth muscle cells. However, there are atypical AMLs composed almost exclusively of epithelioid cells with pronounced abnormal blood vessels and with less or no lipocytes. These atypical AMLs have been referred to as Epi-AML [4]. The appearance of an Epi-AML has been reported in the kidney but rarely in the liver [5]. Epi-HAML is prone to occur in females, without specific symptoms; the tumors were always found incidentally in health examinations. With increasing size of tumor, some patients may show symptoms caused by tumor compression [2, 6].

The imaging characteristics of HAML are correlated with its histological components. The fat content of typical HAMLs produces a characteristic appearance on imaging studies, thereby enabling the preoperative differentiation of typical HAMLs from other hepatic tumors [7]. Previous literature showed most Epi-HAML tumors were completely devoid of adipose tissue, so little or no fatty attenuation or tense was observed on CT or MRI images, which is a characteristic radiographic features of Epi-HAML compared with that of typical HAML. However, the low fat content of Epi-HAML makes the diagnosis of these tumors difficult [1, 2, 4].In our study, precontrast CT or MRI did not detect fatty attenuation in any of the tumors, a result consistent with previous reports.

On dynamic CT or MRI studies, all the tumors were markedly enhanced in the arterial phase, suggesting that Epi-AML is a hypervascular tumor. Most lesions (four) decreased obviously in the portal venous/delayed phase. These findings are compatible with hepatocellular carcinoma (HCC) [8]. The distinction between these lesions by enhancement pattern can be difficult. However, abundant central vessels, especially thickly distorted vessels were observed in these lesions, which is a characteristic radiographic features of Epi-HAML compared with other hypervascular hepatic tumors [9]. The other two lesions with small or no vessels continued enhanced in the portal venous/delayed phase. These lesions with prolonged enhancement pattern should be differentiated from HCCs and cavernous hemangiomas [10].

In our study, discrete capsule enhancement was observed within one lesion on contrast MRI. According to the pathology specimens and the literature [11], there is no true capsule in Epi-AML, but in some large lesions, intact or discrete pseudocapsule enhancement can be observed, composed of the compressed liver parenchyma and sparse fibrosis tissues with small vessels, resulting in delayed enhancement on late phase. Additionally, a capsule can be found in most HCCs(~70 % of HCCs with a diameter >2 cm are encapsulated), so the margins of HCC are more clear than those of Epi-AML in the portal venous phase, a suggestive finding for correct diagnosis.

Although typical AMLs were regarded as universally benign tumors and often grow slowly, it is becoming increasingly clear that Epi-AML should be regarded as tumors of uncertain malignant potential. Recently, rare cases of Epi-AML with tumor recurrence, and vascular invasion were reported [12, 13]. Surgical resection should be considered for all symptomatic patients. Conservative management with close follow-up is suggested in asymptomatic patients with small mass (<5 cm) that are diagnosed through fine-needle aspiration biopsy [14].

In summary, Epi-AML usually presents as a well-defined, low fat content and unencapsulated mass in an asymptomatic woman. In addition, hypervascularity with central punctiform or filiform vessels is a characteristic radiographic features for Epi-HAML. The enhancement pattern is divided into two types, lesions with abundant central vessels rapidly decreased and lesions with small or no vessels demonstrated prolonged enhancement in the portal venous/delayed phase.
